# Emotion recognition in borderline personality disorder: effects of emotional information on negative bias

**DOI:** 10.1186/s40479-015-0031-z

**Published:** 2015-06-26

**Authors:** Sabrina Fenske, Stefanie Lis, Lisa Liebke, Inga Niedtfeld, Peter Kirsch, Daniela Mier

**Affiliations:** Central Institute of Mental Health, University of Heidelberg / Medical Faculty Mannheim, J5 68159 Mannheim, Germany

**Keywords:** Borderline personality disorder, Emotion recognition, Affect processing, Facial affect, Social cognition, Negative bias, Emotion regulation, Context

## Abstract

**Background:**

Borderline Personality Disorder (BPD) is characterized by severe deficits in social interactions, which might be linked to deficits in emotion recognition. Research on emotion recognition abilities in BPD revealed heterogeneous results, ranging from deficits to heightened sensitivity. The most stable findings point to an impairment in the evaluation of neutral facial expressions as neutral, as well as to a negative bias in emotion recognition; that is the tendency to attribute negative emotions to neutral expressions, or in a broader sense to report a more negative emotion category than depicted. However, it remains unclear which contextual factors influence the occurrence of this negative bias. Previous studies suggest that priming by preceding emotional information and also constrained processing time might augment the emotion recognition deficit in BPD.

**Methods:**

To test these assumptions, 32 female BPD patients and 31 healthy females, matched for age and education, participated in an emotion recognition study, in which every facial expression was preceded by either a positive, neutral or negative scene. Furthermore, time constraints for processing were varied by presenting the facial expressions with short (100 ms) or long duration (up to 3000 ms) in two separate blocks.

**Results:**

BPD patients showed a significant deficit in emotion recognition for neutral and positive facial expression, associated with a significant negative bias. In BPD patients, this emotion recognition deficit was differentially affected by preceding emotional information and time constraints, with a greater influence of emotional information during long face presentations and a greater influence of neutral information during short face presentations.

**Conclusions:**

Our results are in line with previous findings supporting the existence of a negative bias in emotion recognition in BPD patients, and provide further insights into biased social perceptions in BPD patients.

**Electronic supplementary material:**

The online version of this article (doi:10.1186/s40479-015-0031-z) contains supplementary material, which is available to authorized users.

## Background

Borderline Personality Disorder (BPD) can be characterized by severe emotional dysregulation and affective instability [1]. Patients suffering from BPD show a significant fear of being abandoned and pervasive problems in interpersonal relationships [2, 3]. One possible cause for these frequently occurring interpersonal conflicts might be a misattribution of social signals: Patients with BPD demonstrate a more negative and hostile perception of social relationships [4], are characterized by an anxious attachment style [5], and judge others as more negative, rejecting and aggressive (e.g. [6–8] see also [9] for a review). However, previous studies directly investigating emotion recognition in BPD provide heterogeneous results, ranging from deficits to a heightened sensitivity for emotional expressions [9, 10]. Hence, it can be assumed that the emotion recognition performance in BPD is subject to influencing factors, such as the emotional context [11].

Most of the early studies on emotion recognition in BPD reported deficits in emotion recognition, particularly in the identification of negative emotions [12–16]. A recent meta-analysis however [10], reported that BPD patients show an overall deficit in recognition accuracy (when including all basic emotions and neutral expressions in the analysis). Furthermore, this meta-analysis suggests that BPD patients are not impaired in the recognition of all negative emotions, but have a specific deficit in the recognition of disgust and anger. However interestingly, the largest deficit was revealed for the identification of neutral facial expressions, suggesting that BPD patients tend to misattribute emotions to faces that do not convey emotional information. In line with this meta-analysis of Daros and colleagues [10], another recent meta-analysis of Mitchell and colleagues [17] supports the assumption of a negative bias in BPD; i.e. that patients with BPD demonstrate a tendency to attribute negative emotions to neutral facial expressions.

In agreement with the idea that patients with BPD do not show profound deficits in the recognition of negative emotions, but rather a negative bias, there are several studies reporting either no significant emotion recognition deficit in BPD at all [18, 19] or a deficit that occurs only under specific conditions. In some of these studies, difficulties in emotion recognition were only revealed by low intensity levels of emotional expression [20], or when a fast discrimination was required [21]. Furthermore there are studies that demonstrate higher accuracy in the classification of emotional expressions in BPD [22, 23]. Wagner and Linehan [23] for example, reported a heightened sensitivity in the recognition of fearful facial expressions only, and Lynch and colleagues [22] showed that BPD patients tend to identify happy and angry faces at an earlier level of intensity. For male faces with an angry expression this was also true in a study by Veague and Hooley [24]. In addition, there are findings explicitly pointing to a response bias in BPD patients favoring negative emotion categories when confronted with ambiguous or neutral facial expressions [21, 24–27]. For ambiguous expressions (morphing from one emotion to another), BPD patients had a response bias, favoring anger over disgust and happiness [25]. Among the studies using continuously morphed pictures (morphing from neutral to a full emotional display), several found no differences in recognition threshold between groups [18, 25, 26], but higher error rates for fear and surprise in one of the studies [26]. In addition, Veague and Hooley [24], found not only that patients with BPD had a higher sensitivity for male faces that displayed anger, but also a response bias for anger in neutral faces and morphed faces that contained no anger-cues (happy and fearful). In contrast to these studies pointing to a negative bias, a recent study by Daros and colleagues [28] suggested that a misattribution of emotional states may be linked to both a misinterpretation as negative as well as positive valent emotional states; i.e. a general tendency to attribute emotions to neutral facial expressions. Taken together, albeit not all individual studies found a significant negative bias in BPD (e.g. [28]), recent meta-analytic evidence suggest a negative response bias to neutral and ambiguous expressions [17] that might be pronounced for the misattribution of anger [24, 25]. However, it is not clear why this deficit in the recognition of neutral as well as emotional facial expressions and especially the negative bias is not found consistently across studies.

One explanation is that when asking for basic emotions, a statistical bias for negative emotions is inherent. Another explanation is that emotion recognition performance in BPD is depending on the context and modulated by the prominent emotion regulation deficits in this patient group [14].

Patients with BPD are known to experience frequent states of negative emotions and aversive tension [29, 30]. This affective instability seems to arise from a high susceptibility for emotional information in combination with a severe emotion regulation deficit [31]. It was shown in healthy participants that negative affect biases the processing of emotional information [32]. Mobbs and colleagues [11] showed that preceding emotional information shifted ratings for identical faces in the direction of the preceding emotional information (see also [33, 34]). Moreover, studies using emotional contextual information found that emotion recognition performance was biased by this contextual information (e.g. [11, 35]). Interestingly, in a study with euthymic bipolar patients, it was shown that priming with emotional facial expressions resulted in a negative shift of pleasantness judgments for neutral target faces [36]. Furthermore, Hooker and colleagues [37] demonstrated that negative affective priming with pictures from the International Affective Picture System [38] led to lower trustworthiness ratings of faces in schizophrenia patients than in healthy controls, indicating a higher susceptibility for negative emotional information in this patient group [37]. Hence, there is considerable evidence for an influence of emotional information on emotion perception in healthy people, as well as in clinical samples.

However, to our knowledge - despite the vast evidence of emotion regulation deficits in patients with BPD - until now no comparable study exists that investigates the influence of emotional information on emotion recognition, and/or the association between emotion recognition and emotion regulation in BPD.

Another factor that affects emotion recognition performance is the available time to perceive and process the incoming information. Several authors showed that longer presentation times increased discrimination performance in healthy samples (e.g. [39–41]). However, studies on emotion recognition in BPD differ in regard to the given time constraints. So far there are no studies that systematically investigated the effect of this factor. The first study emphasizing the important role of processing time for emotion recognition in BPD patients was conducted by Dyck and colleagues [21]. The authors demonstrated that fast emotion discrimination leads to higher arousal levels and more errors in emotion recognition in BPD patients than in healthy controls. In this case, particularly, neutral facial expressions were more often identified as negative.

Therefore, the aim of the present study was to investigate the influence of emotional information on emotion recognition performance in BPD. We hypothesized that (1) patients with BPD show a deficit in emotion recognition compared to healthy participants. We further assumed (2) that this deficit is augmented when facial expressions are preceded by emotional information (i.e. that patients with BPD perform worse than healthy control participants when the preceding information is arousing and has an emotional valence in comparison to emotionally neutral preceding information). Since it was shown that time pressure causes an increase in arousal levels and results in stronger negative bias in BPD [21], the influence of emotional information on emotion recognition in BPD was assessed with and without time pressure. It was hypothesized that (3) restricted presentation time of the facial expression leads to a pronounced influence of the emotional information on emotion recognition. Moreover for neutral facial expressions, we expected that (4) the emotion recognition deficit in BPD is due to a negative bias. Lastly, it was hypothesized that (5) the negative bias is associated with self-reported deficits in emotion regulation.

## Methods

### Sample

Before participating in the study, participants were informed about study procedures and gave written informed consent. The study was approved by the local Ethics Board of the Medical Faculty Mannheim, University of Heidelberg.

The sample consisted of 32 females with BPD and 31 healthy female controls (Table 1). All patients met DSM-IV criteria for BPD [42]. 93.75 % of them also had a comorbid psychiatric diagnosis, and 75 % received psychotropic medication (see Additional file 1: Table S1 for percentages of specific diagnoses and medication). Diagnoses were made by experienced clinicians (psychologists or psychiatrists) at the Outpatient Unit of the Clinic for Psychosomatics and Psychotherapeutic Medicine at the Central Institute of Mental Health (CIMH) by means of a German version of the SCID-I interview [43], and the International Personality Disorder Examination (IPDE; [44]). Patients with a comorbid diagnosis of schizophrenia, bipolar disorder, or addiction (currently or within the last 3 years), as well as with neurological diseases were excluded. Sixteen of the patients were inpatients. Healthy controls were recruited via local databases of the CIMH and participated in the SCID-I interview and completed the SCID-II questionnaire [45] to exclude participants with current or life-time psychiatric diagnosis. Moreover, healthy participants were excluded when reporting a neurological disorder. General inclusion criteria were the ability to give written informed consent and sufficient command of the German language to understand task instructions and to complete the questionnaires.

**Table 1:** Sample characteristics

	BPD *N = 32*	HC *N = 31*	*p*
Mean age in years	30.35 *(8.22)*	29.84 *(7.70)*	0.838
Mean years of education	11.03 *(1.64)*	11.52 *(1.57)*	0.235
DERS sum score	128.10 *(24.38)*	59.77 *(11.86)*	<0.001
BSL-23 sum score	2.24 *(0.79)*	0.15 *(0.19)*	<0.001
PANAS_positive_pre	2.43 *(0.64)*	2.95 *(0.60)*	0.002
PANAS_positive_post	2.07 *(0.62)*	2.51 *(0.71)*	0.012
PANAS_negative_pre	1.97 *(0.73)*	1.07 *(0.08)*	< .001
PANAS_negative_post	2.06 *(0.79)*	1.10 *(0.19)*	<0.001

*Note:* Means and standard deviations (in parentheses) of *DERS* = Difficulties in Emotion Regulation Scale, *BSL-23* = Borderline Symptom List-23, *PANAS* = Positive and Negative Affect Schedule, positive_pre = positive affect before the experiment, positive_post = positive affect after the experiment, negative_pre = negative affect before the experiment, negative_post = negative affect after the experiment

After participating in the experiment, all participants completed several questionnaires. Severity of borderline symptoms and emotion regulation deficits were assessed with the Borderline Symptom List-23 (BSL-23; [46, 47]) and the Difficulties in Emotion Regulation Scale (DERS; [48]). The current affective state was assessed before and after the experiment with the Positive and Negative Affect Schedule (PANAS; [49, 50]). Concerning the PANAS and BSL-23, data of two patients and one healthy control is missing. Further, data of the DERS is missing for three patients and one healthy control (see Table 1 for group averages).

### Emotion recognition task

An emotion recognition task was applied, in which each facial expression was preceded by a picture varying in valence and arousal. The preceding pictures were taken from the International Affective Picture System (IAPS; [38]). The IAPS pictures either showed a scene with positive valence and high arousal (e.g. depicting sport scenes), negative valence and high arousal (e.g. depicting crime scenes) or neutral valence and low arousal (e.g. depicting daily conversational situations). Importantly, we explicitly avoided the selection of pictures with a sexual theme for the positive IAPS category to prevent adverse responses in the BPD patients that are due to a history of sexual traumatization. Valences of the positive and negative pictures were matched to be equally distant from the neutral pictures (positive: *M* = 7.06, *SD* = 0.52; neutral: *M* = 5.02, *SD* = 0.36; negative: *M* = 3.02, *SD* = 0.45). Positive and negative pictures were also matched for arousal extent (positive: *M* = 5.98, *SD* = 0.52; negative: *M* = 5.99, *SD* = 0.47) while neutral pictures had a lower arousal (*M* = 3.09, *SD* = 0.37; see Additional file 1: Table S2 for a list of all IAPS pictures that were presented in the course of the experiment). The facial stimuli were taken from the “NimStim Set of Facial Expressions” [51] and consisted of 5 male and 5 female actors. The faces showed an emotional (happy or angry) or neutral expression. To avoid ceiling effects in emotion recognition performance, emotional facial expressions with reduced emotion intensity were applied (60 % emotion, 40 % neutral). The morphed facial expressions were taken from Matzke and colleagues [18]. Participants were instructed to look at all pictures, but to rate the valence of the facial expressions only, and not the valence of the scenes, by selecting one of three buttons (positive, neutral, negative) on a standard computer keyboard. We decided using only three emotion categories that were presented with equal probability to avoid a statistical bias for the selection of a negative emotion that is merely due to the presence of more negative categories; i.e. as it naturally occurs when using all basic emotions.

**Fig. 1:**
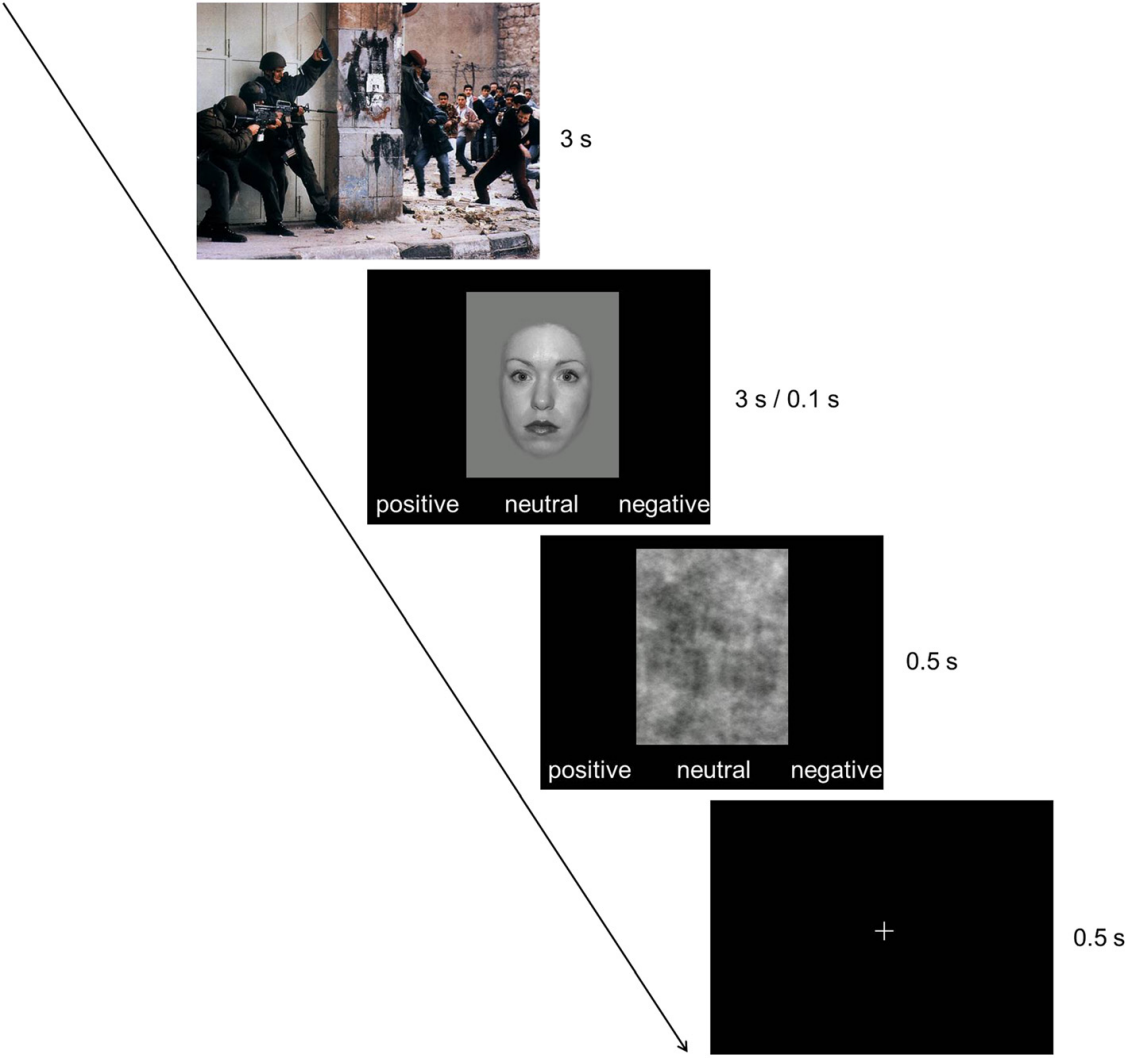
Experimental design with up to 3 seconds presentation of the facial expression in the “self-paced” condition and 100 milliseconds presentation of the facial expression in the “timed” condition

The task was applied in two blocks, differing in the presentation time of the facial expression. In both blocks, IAPS pictures were shown for 3 seconds and were immediately followed by a picture with a facial expression (Fig. 1). In one of the blocks, the facial expressions were presented until one of the response buttons was pressed, but for 3 seconds at most (“self-paced” condition). In the other block, presentation time was restricted to 100 milliseconds (“timed” condition). In both blocks, participants had up to 3 seconds to rate the valence of the emotion, and the facial expression was followed by a mask (a grey rectangle) for 500 milliseconds. Trial order was pseudo-randomized and block order was counterbalanced across participants. Each block consisted of 90 trials, i.e. 10 combinations of each IAPS category (positive, neutral, negative) with each face category (happy, neutral, angry) and took about 11 minutes. While completing the emotion recognition task, galvanic skin response and heart rates were recorded. The results from this psychophysiological assessment will be reported elsewhere.

### Rating of experimental stimuli

Immediately after the emotion recognition task, the applied pictures were presented again for valence and arousal ratings. This additional evaluation of stimulus valence and arousal was completed to assess ratings without an influence of the experimental setup. For this purpose, faces and scenes were presented to the participants in two separate blocks, always starting with the faces block. Participants were asked to indicate the valence and arousal of each of the pictures using the Self-Assessment-Manikin (SAM) [52, 53] on a 5-point scale. This rating procedure was self-paced.

## Results

Statistical analyses were performed with SPSS Statistics 21 (IBM Corporation, New York). Applying one-sample Kolmogorov-Smirnov-tests, no significant violations of the normal distribution were revealed (all *p*s > 0.11). In the case that Levene-tests for equality of variances revealed significant differences in variance between groups, the according *p*-statistics are reported with Greenhouse-Geisser correction. Effect sizes are specified as Cohen’s *f* and *d*.

### Emotion recognition task

#### Hypothesis 1

**Fig. 2:**
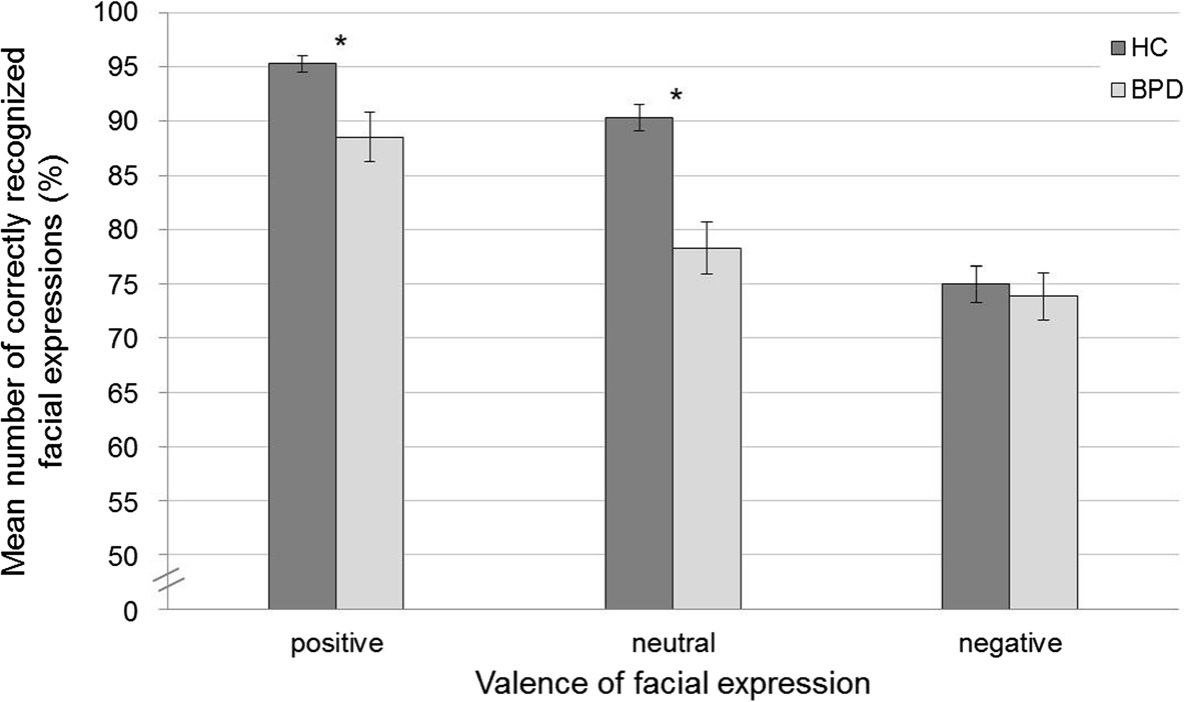
Mean numbers of correctly recognized facial expressions in percent correct, separated for group and face valence. Error bars display the standard errors, stars indicate significant group differences (*p* < 0.05)

To investigate the first hypothesis, that patients with BPD show impaired emotion recognition performance, a 2 (group) × 3 (face valence) repeated measures ANOVA was conducted (Table 2). There was a significant face valence × group interaction: Post hoc comparisons revealed that BPD patients identified both neutral and positive facial expressions less often correctly than healthy controls (neutral: *t* (61) = 4.52, *p* < 0.001, *d* = 1.19; positive: *t* (61) = 2.80, *p* = 0.008, *d* = 0.79), but not negative ones (*t* (61) = 0.42, *p* = 0.678, *d* = 0.11) (see Fig. 2). Due to the higher-order interaction effect, the interpretability of the main effect of group is restricted. However, there was also a main effect of face valence: Positive facial expressions were better recognized than neutral and negative facial expressions (neutral: *t* (62) = 4.96, *p* < 0.001, *d* = 0.62; negative: *t* (62) = 9.86, *p* <0.001, *d* = 1.24). Further neutral facial expressions were more often recognized correctly than negative facial expressions (*t* (62) = 4.19, *p* < 0.001, *d* = 0.53).

**Table 2:** (a) Statistical data of the group × face valence repeated measures ANOVA for emotion recognition performance, and (b) descriptive values for the percentages of correctly recognized facial expressions

a)				
	*df*	*F*	*f*	*p*
Group	1,61	19.32	0.65	<0.001
Face valence	2,122	44.21	1.12	<0.001
Group × face valence	2,122	4.27	0.27	0.023
b)				
	BPD		HC	
Valence of facial expression	*M*	*SD*	*M*	*SD*
Positive	88.54	13.00	95.27	3.97
Neutral	78.28	13.44	90.32	6.74
Negative	73.85	12.26	75.00	9.30

#### Hypotheses 2 and 3

**Fig. 3:**
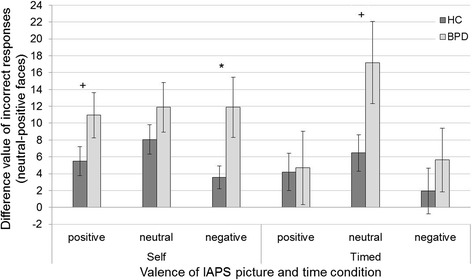
Difference values, showing percent of incorrectly recognized neutral minus incorrectly recognized positive facial expressions, separated for the preceding IAPS picture, time condition and group. Error bars display the standard errors, stars indicate significant group differences (*p* < 0.05), a plus indicates marginally significant group differences (*p* < 0.1)

To further analyze whether the deficits in the perception of positive and neutral facial expressions are influenced by emotional information and time constraints, a group × face valence × IAPS valence × time repeated measures ANOVA was conducted (Table 3). This analysis revealed a marginally significant four-way interaction, indicating that differences between groups were differentially affected for neutral and positive faces by the preceding IAPS picture and by the time constraints. Post-hoc comparisons to disentangle this interaction effect were conducted separately for the two presentation times, as well as for the comparison between the presentation times. There was a stronger effect of preceding negative emotional information on the recognition of neutral versus positive facial expressions in the BPD group compared to healthy controls in the self-paced condition (*t* (61) = −2.17, *p* = 0.034, *d* = −0.60). Moreover, there was a trend for more incorrect responses for neutral compared to positive facial expressions in the BPD group compared to healthy controls when the facial expressions were preceded by positive emotional information in the self-paced condition (*t* (61) = −1.70, *p* = 0.093, *d* = −0.44). In the timed condition, there was a marginally significant higher error rate for neutral compared to positive facial expressions in the BPD group compared to the healthy controls when the preceding information was neutral (*t* (61) = −1.99, *p* = 0.051, *d* = −0,54). These difference values did not differ significantly between the two time conditions (Fig. 3). Due to the higher-order interaction effect, the interpretability of the main effects and lower-order interaction effects is restricted.

**Table 3:** (a) Statistical data of the group × face valence x IAPS valence x time repeated measures ANOVA for emotion recognition performance, and (b) descriptive values for the percentage of incorrectly recognized facial expressions, depending on the IAPS-category and the timing

a)				
	*df*	*F*	*f*	*p*
Group	1,61	18.80	0.64	<0.001
Face valence	1,61	26.49	0.79	<0.001
IAPS valence	2,122	3.54	0.25	0.032
Time	1,61	43.87	1.09	<0.001
Group × Face valence	1,61	3.32	0.24	0.073
Group × IAPS	2,122	0.59	0.1	0.557
Group x time	1,61	0.19	0.05	0.664
Face valence × IAPS valence	2,122	6.97	0.36	0.010
Face valence × time	1,61	1.02	0.13	0.317
IAPS valence × time	2,122	0.10	0.04	0.902
Group × face valence × IAPS valence	2,122	1.07	0.13	0.346
Group × face valence × time	1,61	0.053	0.03	0.819
Group × IAPS valence × time	2,122	0.32	0.07	0.727
Face valence × IAPS valence × time	2,122	2.35	0.20	0.099
Group × face valence × IAPS valence × time	2,122	2.49	0.21	0.087
b)				
	BPD		HC	
Incorrect responses	*M*	*SD*	*M*	*SD*
*Self-paced*				
Positive IAPS				
Neutral face	15.31	16.06	5.81	9.58
Positive face	4.38	8.40	0.32	1.80
Neutral IAPS				
Neutral face	19.06	15.32	8.71	10.56
Positive face	7.19	15.08	0.65	2.50
Negative IAPS				
Neutral face	18.75	19.80	4.84	8.51
Positive face	6.88	13.06	1.29	4.28
*Timed*				
Positive IAPS				
Neutral face	20.94	19.73	11.61	9.69
Positive face	16.25	16.61	7.42	7.73
Neutral IAPS				
Neutral face	29.06	22.91	14.52	10.60
Positive face	11.88	14.47	8.06	9.46
Negative IAPS				
Neutral face	23.13	18.22	11.61	11.28
Positive face	17.50	19.84	9.68	11.40

#### Hypothesis 4

**Fig. 4:**
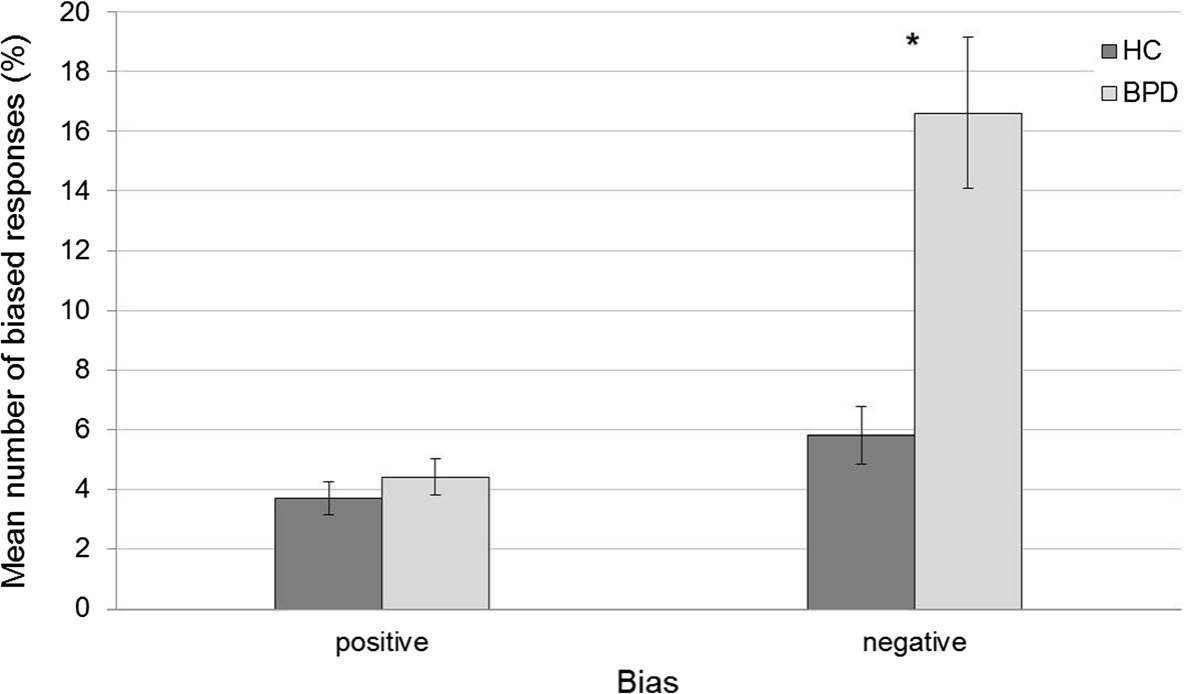
Bias × group interaction. Percentages of all responses to neutral facial expressions that were either positively or negatively biased, separated for the two groups. Error bars display the standard errors, stars indicate significant group differences (*p* < 0.05)

To examine whether the emotion recognition deficit for neutral facial expressions in the BPD patients was due to a negative bias, the incorrect answers in response to neutral facial expressions were sub-divided in negatively and positively biased responses, i.e. a misattribution of a positive or negative valence. A 2 (group) × 2 (bias valence) repeated measures ANOVA was conducted (Table 4). There was a significant group × bias interaction: Post hoc comparisons revealed that BPD patients showed a stronger negative bias than healthy controls (*t* (61) = −3.98, *p* < 0.001, *d* = −1.09), while groups did not differ in the amount of positive bias (*t* (61) = −0.882, *p* = 0.381, *d* = −0.22) (see Fig. 4). Due to this higher-order interaction effect, the interpretability of the main effects of group and bias valence is restricted.

**Table 4:** (a) Statistical data of the group × bias valence repeated measures ANOVA for the recognition of neutral facial expressions, and (b) descriptive values for the percentages of biased responses

a)				
	*df*	*F*	*f*	*p*
Group	1,61	18.09	0.62	<0.001
Bias valence	1,61	22.39	0.71	<0.001
Group × Bias valence	1,61	11.17	0.47	0.001
b)				
	BPD		HC	
Valence of bias	*M*	*SD*	*M*	*SD*
Positive	4.43	3.40	3.71	3.03
Negative	16.61	14.38	5.81	5.34

Additional exploratory comparisons for the amount of negative bias between the three most common comorbidities in our BPD-sample, as well as between BPD-in- and BPD-outpatients were not significant (depression: *p* = 0.615, PTSD: *p* = 0.700, eating disorders, *p* = 0.181, inpatients: *p* = 0.324).

#### Hypothesis 5

To analyze hypothesis 5; i.e. the association between emotion regulation abilities and the negative bias, BSL- and DERS-scores were analyzed. Pearson correlation coefficients of the questionnaire data and the amount of negative bias were calculated for all participants. There were significant correlations of the BSL-score (*r* = 0.545, *p* < 0.001) and the DERS-total score (*r* = 0.606, *p* < 0.001) with the negative bias across all participants. In the control group, BSL-scores correlated significantly with the amount of negative bias (*r* = 0.629, *p* < 0.001), while this correlation was not significant in the BPD group (*r* = 0.287, *p* = 0.125). Further, correlations were only trend-level significant in the control group between the negative bias and the DERS-sum score (*r* = 0.347, *p* = 0.060), while in the BPD group, there was a significant correlation between the negative bias and the DERS-sum score (*r* = 0.453, *p* = 0.014).

### Ratings of the experimental stimuli

The analysis of the SAM ratings showed no group differences for valence ratings. All participants rated positive scenes and positive faces with higher and negative ones with lower valence than neutral pictures. Overall, negative IAPS pictures were rated with highest arousal. Positive IAPS pictures were rated with significantly lower arousal and neutral IAPS pictures with the lowest arousal. For neutral, negative and on a trend-level for positive facial expressions, higher arousal ratings were found in the BPD group. Arousal ratings were also higher in the BPD group than in the control group for the IAPS pictures. Arousal and valence ratings, as well as according analyses are reported in the Additional file 1.

## Discussion

To investigate the influence of emotional information on emotion recognition in BPD, an emotion recognition task in which each facial expression was preceded by an IAPS picture, varying in valence and arousal, was applied. It was hypothesized that patients with BPD show an emotion recognition deficit and that this deficit is augmented when facial expressions are preceded by emotional information, and when processed under time constraints. Furthermore, it was assumed that the emotion recognition deficit for neutral faces in BPD patients is due to a negative bias, which in turn is associated with emotion regulation deficits.

In accordance to our hypothesis, BPD patients showed a clear emotion recognition deficit that was evident for neutral and positive facial expressions. Moreover, this deficit was accompanied by a negative bias in the perception of neutral faces. These results are in line with findings indicating that patients with BPD have the most pronounced difficulties in the classification of neutral facial expressions [10], as well as a negative bias [17]. Interestingly and in contrast to previously reported higher error rates for BPD patients in the identification of negative emotions [12–14, 16], we found a comparable performance in the recognition of angry facial expressions as negative valent. Hence, our results suggest that BPD patients do not have a general emotion recognition deficit, but a deficit in the recognition of emotions *without* a negative valence. Alternatively, with regard to different negative emotions, especially the ability to recognize anger, might be spared in BPD. For example, Guitart-Masip and colleagues [13] also showed no group differences for the recognition of angry facial expressions, as used in our paradigm as well, but for disgusted and fearful faces. From a neurobiological point of view, the often reported increased amygdala-activation in BPD patients in response to facial expressions [19, 54, 55] might elicit a higher vigilance per se and especially a higher vigilance for threatening information, making BPD patients even more sensitive for angry facial expressions. Hence, threatening information might be more salient and subjectively more likely to occur to patients with BPD, leading to a “more accurate” recognition when an angry face is presented, but to more false positive responses when an expression is not negative, particularly not angry [16]. This proneness to false positives might be enhanced by the severe emotion regulation deficits in BPD [2, 56]. In BPD patients’ daily life, this might lead to the often occurring negative expectations concerning others (e.g. [6–8]).

As mentioned before, on a neurobiological level, several authors [19, 54, 55] showed an enhanced amygdala-activation in patients with BPD in response to facial expressions. This limbic hyperactivation occurs in concert with deficits in the regulatory function of the prefrontal cortex (PFC, particularly the anterior cingulate cortex) [55, 57]. Interestingly, a recent meta-analysis by Ruocco and colleagues [57] additionally found evidence for insula hyperactivation in response to negative in comparison to neutral stimulus materials in BPD. The authors interpret this insula hyperactivation as possibly underlying the intensified subjective experience of negative emotions in BPD. With the insula as a connecting region between frontal and subcortical brain regions, this hyperactivation also supports the assumption of impaired fronto-limbic regulation of negative emotions in BPD. To our knowledge, not explicitly in BPD [58], but in other disorders, this reduced control of the PFC over the limbic system has been repeatedly shown to be associated with deficits in emotion regulation [59–61]. In the context of social cognition, reduced control of the PFC over the amygdala might enhance the tendency to categorize neutral (and maybe also positive) stimuli as more negative [27]. Hence, a deficit in emotion regulation should be associated with a more pronounced negative bias. Indeed, we found that the number of negatively biased responses was significantly correlated with emotion regulation deficits measured by the DERS across all participants, and within the BPD patient group. There was only a significant correlation with the strength of borderline symptoms measured by the borderline symptom list (BSL-23) in the healthy group, but not for the BPD patients. Accordingly, it can be concluded that deficits in emotion regulation are associated with the amount of negative bias in general, while the BSL (which serves as a more global measurement of emotion regulation deficits and borderline symptom severity) might only significantly explain variance below a specific cut-off (i.e. within a non-clinical range of occurrence).

In addition to deficits in emotion regulation, other factors might influence emotion recognition performance in BPD. Considering previous findings from studies investigating the influence of emotional context information [11, 33–35] and priming [36, 37] on emotion recognition, we hypothesized that emotional information that precedes emotion recognition should impair the performance in BPD patients. It was further assumed that time restriction should enhance this effect of emotional information in BPD patients. This assumption was based on the study by Dyck and colleagues [21] who failed to show a general emotion recognition deficit in BPD, but reported a deficit in a fast emotion discrimination task. In particular, it was assumed that a brief presentation time of the faces forces more intuitive emotion recognition and in consequence might result in a stronger influence of the preceding IAPS picture.

We found a marginally significant interaction of IAPS valence, time condition, face valence and group. In the condition without time restriction, negative emotional information was associated with more errors in the recognition of neutral facial expressions compared to positive expressions in the BPD group than in the control group. This was also (on a trend-level) true for positive emotional information. Hence, this provides first evidence for the assumption that emotional information influences emotion recognition performance in BPD to a higher extent than in healthy controls and that this is especially true for the recognition of neutral facial expressions. Interestingly, in the condition with time restriction, there were more errors for neutral in comparison to positive facial expressions in the BPD group compared to healthy controls when the preceding information was neutral. Thus, in the case of limited processing time of the facial information, especially neutral information seems to elicit false responses to neutral facial expressions. One explanation for that might be that neutral information is more ambiguous for patients with BPD, and in consequence is not perceived as neutral, especially when processed under time pressure. This perceived ambiguity could be augmented and results in misinterpretations when the target is also not showing an emotional valence. Taking into account that post hoc valence ratings of the IAPS pictures and the facial expressions did not differ significantly between the groups (see Additional file 1: rating of stmuli), it is remarkable that emotion recognition was more impaired and more negatively biased in the BPD group when it was combined with preceding information. Hence, it can be assumed that the experimental pairing of IAPS pictures with facial expressions fostered the emotion recognition deficit in BPD patients.

However, it has to be acknowledged that the four-way interaction including the IAPS valence and time constraints was only marginally significant. Not disregarding the reduced statistical power of this four-way ANOVA, an explanation might be the occurrence of carry over effects resulting from the pseudo-randomized presentation of the different emotional categories. Indeed, all participants showed less positive affect after the experimental task (see Additional file 1: affective state). In agreement with our assumption, there was a significant correlation between the increase of negative affect in the course of the experiment and the amount of negative bias (see Additional file 1: correlations of negative bias with affective state), which again emphasizes current mood as an influencing factor for emotion recognition. It is important to mention that the applied IAPS pictures were selected to be appropriate for a sample of female BPD patients. Hence, due to the high prevalence of sexual traumatization in BPD [62, 63], no pictures depicting sexual scenes were used for the IAPS category with positive valence. In consequence, to match for arousal in the positive and the negative category, only pictures of average arousal and thus also average valence levels could be applied in both categories, which might have reduced the influence of the preceding emotional information. Future studies might use a blockwise presentation of the different preceding valences or a mood induction to investigate whether a stronger differential influence of emotional information is elicited when carry-over effects can be excluded. Furthermore, it would be interesting to disentangle stimulus valence and arousal to investigate the effect of these dimensions on emotion recognition in BPD. The dependency of valence and arousal in our study is due to the fact that they represent different parameters of motivational systems: While the valence dimension indicates *which system* is activated (appetitive or aversive), arousal shows to *which degree* the system is activated [64]. Hence, valence and arousal ratings are highly correlated for the IAPS pictures [64], and it would be interesting to develop novel paradigms with other stimulus materials/arousal induction methods that allow to investigate whether the activation of the aversive system or the degree of activation - independent of the system - is more important for social-cognitive performance in BPD.

A limitation of the current experimental design can be seen in the categorical response alternatives: False responses for positive facial expressions per se were negatively biased and for negative facial expressions per se were positively biased. Therefore, future studies might additionally include a response format that allows for shifting responses within one category by applying continuous response formats. Moreover, albeit we carefully matched the emotional IAPS pictures for the normative arousal levels provided with the IAPS database, participants in our study rated IAPS pictures with a negative valence with higher arousal levels than the ones with a positive valence. Hence, the potential influence of positive IAPS pictures was weaker than intended and has to be interpreted with care. However, Hooker and colleagues [37] did not find a priming effect of positive IAPS pictures on trustworthiness ratings either, possibly suggesting a stronger influence of negative than positive emotional information on social cognition. Moreover, since the study was of an exploratory nature, to investigate the complex interaction between different emotional information, emotion recognition categories and processing time in BPD for the first time, no experiment-wise error correction was applied. Thus, future studies are needed that replicate our findings, probably using paradigms with a more ecological experimental design.

## Conclusions

In conclusion, the study replicated previous findings of an emotion recognition deficit for neutral and positive facial expressions in BPD patients. In addition, we could show a differential influence of valence of the preceding information and processing time: The emotion recognition deficit for neutral facial expressions was augmented in the BPD group when faces were presented after emotional information, when processing time of the preceding information was not restricted, and after neutral information, when processing time was restricted. While previous studies revealed heterogeneous results concerning the existence of a negative bias in emotion recognition in BPD, our findings provide clear evidence for a negative bias. We suggest that this negative bias in emotion recognition forms a basis for the more negative judgments of others in BPD (e.g. [6–8]). Moreover, we propose that current mood states can influence the social perception of patients with BPD and with this might explain the misperceptions of social signals in social interactions. This negative bias can significantly impair the quality of social interactions and the stability of social bonds. Hence, psychotherapeutic interventions should focus on training patients with BPD in their ability to consciously perceive the influence of situational factors that could affect their current mood and arousal levels, and by this to enable them to reflect on the potential benevolence of interaction partners. Learning to differentiate between current feelings and newly incoming information could help BPD patients to establish more adequate interpretations and behavioral reactions in social interactions.

## Additional file

Additional file 1: **Supplementary materials.**

## Abbreviations

BPD: Borderline Personality Disorder
CIMH: Central Institute of Mental Health
HC: Healthy Controls
SAM: Self-Assessment-Manikin
SCID: Structured Clinical Interview for DSM-IV
IPDE: International Personality Disorder Examination
BSL-23: Borderline Symptom List-23
DERS: Difficulties in Emotion Regulation Scale
PANAS: Positive And Negative Affect Schedule
IAPS: International Affective Picture System
PFC: Prefrontal Cortex
