# Supplementary material for: Emotion recognition in borderline personality disorder: effects of emotional information on negative bias
Source: Borderline Personal Disord Emot Dysregul. 2015 Jun 26;2:10. doi: 10.1186/s40479-015-0031-z (PMC4579484; doi:10.1186/s40479-015-0031-z)
Supplement: Supplementary file 1 — Supplementary materials. [file 40479_2015_31_MOESM1_ESM.docx]

*Supplement*

Table S1 Comorbid psychiatric diagnoses and psychotropic medication of BPD patients

| Comorbid psychiatric diagnosis | 93.75 % |
| --- | --- |
| *Recurrent depressive disorder (of these remitted)* | *66.7 % (10.0%)* |
| *Posttraumatic stress disorder* | *46.7 %* |
| *Eating disorders* | *43.3 %* |
| *Alcohol or Cannabis abuse* | *20.0 %* |
| *Social phobia* | *13.3 %* |
| *Attention deficit hyperactivity disorder* | *10.0 %* |
| *Specific phobia* | *6.7 %* |
| *Panic disorder* | *6.7 %* |
| *Generalized anxiety disorder* | *6.7 %* |
| *Adaptation disorder* | *6.7 %* |
| *Dysthymia* | *3.3 %* |
| *Obsessive-compulsive disorder* | *3.3 %* |
| *Somatoform pain disorder* | *3.3 %* |
| Psychotropic medication | 75.0 % |
| *Antidepressants* | *91.7 %* |
| *Antipsychotics* | *45.8 %* |
| *Sedativa* | *20.8 %* |
| *Methylphenidate* | *12.5 %* |
| *Anticonvulsants* | *8.3 %* |
| *Anti-epileptics* | *4.2 %* |
| *Opioid antagonists* | *4.2 %* |

*Note.* Italic typed diagnoses and medication indicate the sub-proportion of patients having this specific diagnosis or taking this medication, of all patients having any comorbid diagnosis or taking any psychotropic medication.

Table S2 IAPS-codes for the pictures used as emotional information cues in the emotion recognition task

| Positive pictures | Neutral pictures | Negative pictures |
| --- | --- | --- |
| 1650  2303  5260  5480  5621  8031  8170  8260  8400  8500  2216  5622  5623  7501  7502  8034  8179  8191  8200  8496  2345  5626  5629  8210  8250  8300  8370  8467  8490  8499 | 2393  2579  2870  5390  5731  7037  7038  7041  7234  9700  2191  2396  2440  2580  2595  2880  5120  5510  7000  7493  2038  2102  2235  2383  2480  5740  7034  7036  7130  7180 | 2661  2683  2688  2691  3216  3500  6211  6213  8485  9925  1932  6244  6550  6821  6836  6940  8480  9050  9427  9520  5971  6250  6838  9160  9424  9429  9495  9621  9622  9630 |

*Affective state*

To explore the affective state before and after the experiment pre- and post-measurement ratings of the PANAS were analyzed by conducting a 2 (group) x 2 (time) x 2 (PANAS) repeated measures ANOVA. There was a significant group x PANAS interaction: BPD patients showed a lower positive affect (*t*(61) = 3.22, *p* = 0.002, *d* = 0.82), and a higher negative affect compared to healthy controls (*t*(61) = -7.81, *p* < 0.001, *d* = -2,41). Further there was a significant time x PANAS interaction: Post hoc comparisons revealed that participants had higher positive affect before the experiment (Pre: *M* = 2.69, *SD* = 0.67, Post: *M* = 2.29, *SD* = 0.70, *t*(62) = 5.98, *p* < 0.001, *d* = 0.75). Negative affect did not differ between the two assessment time points (Pre: *M* = 1.53, *SD* = 0.69, Post: *M* = 1.59, *SD* = 0.75, *t*(62) = -0.89, *p* = 0.378, *d* = 0.11). However, no group x time x PANAS interaction occurred, which indicates that patients with BPD were not more affected in their mood over the course of the experiment. Due to the higher-order interaction effects, the interpretability of the main effects is restricted (table S3).

Table S3 (a) Statistical data of group

×

time x PANAS repeated measures ANOVA and (b) descriptive values of the PANAS

a)

|  | *df* | *F* | *f* | *p* |
| --- | --- | --- | --- | --- |
| Group | 1,61 | 5.61 | 0.32 | 0.021 |
| Time | 1,61 | 18.31 | 0.62 | <0.001 |
| PANAS | 1,61 | 99.18 | 2.07 | <0.001 |
| Group x time | 1,61 | 0.69 | 0.11 | 0.409 |
| Group x PANAS | 1,61 | 55.51 | 1.32 | <0.001 |
| Time x PANAS | 1,61 | 17.73 | 0.61 | <0.001 |
| Group x time x PANAS | 1,61 | 0.011 | 0.0 | 0.918 |

b)

|  | BPD | | HC | |
| --- | --- | --- | --- | --- |
| PANAS | *M* | *SD* | *M* | *SD* |
| Positive | 2.25 | 0.56 | 2.73 | 0.61 |
| Negative | 2.02 | 0.67 | 1.08 | 0.10 |

*Correlations of negative Bias with affective state*

Pearson correlation coefficients of the current affective state and the amount of negative bias were calculated for all participants. There were significant correlations of the PANAS-score for negative affect before (*r* = 0.418, *p* = 0.001) and after the experiment with the negative bias (*r* = 0.673, *p* < 0.001), as well as of the difference between the time points (*r* = 0.397, *p* = 0.001), while there was no significant correlation of the negative bias and the PANAS-score for positive affect across the whole sample. In the BPD group there were significant correlations only for the PANAS-score for negative affect after the experiment (*r* = 0.609, *p* < 0.001) and for the difference between time points with the amount of negative bias (*r* = 0.486, *p* = 0.005). Interestingly, correlation analysis also revealed a negative significant correlation of the change in positive affect over the experiment with the amount of negative bias (*r* = -0.354, *p* = 0.047). In the control group there was only a significant correlation of the PANAS-score for negative affect before the experiment with the amount of negative bias (*r* = 0.413, *p* = 0.021).

*Rating of stimuli*

A 2 (group) x 3 (face valence) ANOVA for the face ratings revealed a highly significant main effect of face valence: As expected, positive facial expressions (*M* = 1.67, *SD* = 0.43) were rated with a higher valence than neutral (*M* = 3.07, *SD* = 0.35, *t*(62) = 25.03, *p* < 0.001, *d* = 3.13) and negative facial expressions (*M* = 4.13, *SD* = 0.43, *t*(62) = -34.81, *p* < 0.001, *d* = 4.39). Neutral facial expression had higher valence ratings than negative expressions (*t*(62) = -24.78, *p* < 0.001, *d* = 3.13). There was no significant main effect of group for the valence ratings of the facial expressions, as well as no significant interaction for group face valence (table S4).

Table S4 Statistical data of group x face valence repeated measures ANOVA

|  | *df* | *F* | *f* | *p* |
| --- | --- | --- | --- | --- |
| Group | 1,61 | 0.16 | 0.0 | 0.901 |
| Face valence | 2,122 | 909.08 | 15.36 | <0.001 |
| Group x face valence | 1,61 | 0.46 | 0.08 | 0.634 |

To explore arousal-ratings for the faces, a 2 (group) x 3 (face arousal) ANOVA was conducted. There was a significant main effect of group: BPD patients rated faces with a higher arousal than healthy controls (BPD: *M* = 2.52, *SD* = 0.61, HC: *M* =1.91, *SD* = 0.52; *t*(61) = -4.24, *p* < 0.001, *d* = 1.08). Moreover there was a significant group x face arousal interaction: Ratings for arousal were higher in the BPD group compared to the healthy controls for neutral (*t*(61) = -5.88, *p* < 0.001, *d* = 1.54), and negative faces (*t*(61) = -3.34, *p* = 0.001, *d* = 0.85), and on trend-level for positive faces (*t*(61) = -1.79, *p* = 0.078, *d* = 0.45). Due to this higher-order interaction effect, the interpretability of the main effect of face arousal is restricted (table S5).

Table S5 (a) Statistical data of group x face arousal ANOVA and (b) descriptive values of arousal ratings

a)

|  | *df* | *F* | *f* | *p* |
| --- | --- | --- | --- | --- |
| Group | 1,61 | 17.99 | 0.62 | <0.001 |
| Face arousal | 2,122 | 95.77 | 2.01 | <0.001 |
| Group x face arousal | 2,122 | 4.02 | 0.27 | 0.023 |

b)

|  | BPD | | HC | |
| --- | --- | --- | --- | --- |
| Valence of facial expression | *M* | *SD* | *M* | *SD* |
| Positive | 2.04 | 0.70 | 1.74 | 0.62 |
| Neutral | 2.17 | 0.65 | 1.41 | 0.33 |
| Negative | 3.34 | 0.88 | 2.58 | 0.91 |

For IAPS pictures, a 2 (group) x 3 (IAPS valence) ANOVA revealed a significant main effect of IAPS valence: Positive IAPS pictures were rated with a higher valence (*M* = 1.99, *SD* = 0.44) compared to neutral (*M* = 2.76, *SD* = 0.38; *t*(62) = 14.57, *p* < 0.001, *d* = 1.84) and negative IAPS pictures (*M* = 4.28, *SD* = 0.44; *t*(62) = -27.22, *p* < 0.001, *d* = 3.45), and neutral IAPS pictures with a higher valence than negatives ones (*t*(62) = -26.63, *p* < 0.001, *d* = 3,37). There was no significant main effect of group, as well as no significant interaction of group x IAPS valence (table S6).

Table S6 Statistical data of group x IAPS valence ANOVA

|  | *df* | *F* | *f* | *p* |
| --- | --- | --- | --- | --- |
| Group | 1,61 | 0.58 | 0.08 | 0.450 |
| IAPS valence | 2,122 | 611.09 | 10.48 | <0.001 |
| Group x IAPS valence | 2,122 | 0.72 | 0.03 | 0.931 |

To explore arousal-ratings for the IAPS pictures, a 2 (group) x 3 (IAPS arousal) ANOVA was conducted. There was a significant main effect of group with BPD patients having higher arousal ratings overall (BPD: *M* = 2.67, *SD* = 0.68; HC: *M* = 2.30, *SD* = 0.64; *t*(61) = -2.21, *p* = 0.031, *d* = 0.56), and a main effect of IAPS arousal: Negative IAPS scenes were rated with higher arousal (*M* = 3.48, *SD* = 0.89) compared to positive IAPS scenes (*M* = 2.48, *SD* = 0.89, *t*(62) = -13.08, *p* < 0.001, *d* = 1.65) and neutral IAPS scenes (*M* = 1.52, *SD* = 0.57, *t*(62) = -19.58, *p* < 0.001, *d* = 2.47). Positive IAPS scenes were rated with higher arousal than neutral IAPS scenes (*t*(62) = -10.87, *p* < 0.001, *d* = 1.37). No significant interaction for group x IAPS arousal occurred (table S7).

Table S7 Statistical data of group x IAPS arousal ANOVA

|  | *df* | *F* | *f* | *p* |
| --- | --- | --- | --- | --- |
| Group | 1,61 | 4.88 | 0.29 | 0.031 |
| IAPS arousal | 2,122 | 246.69 | 4.52 | <0.001 |
| Group x IAPS arousal | 2,122 | 0.72 | 0.03 | 0.931 |
